# Blockchain technology for improving clinical research quality

**DOI:** 10.1186/s13063-017-2035-z

**Published:** 2017-07-19

**Authors:** Mehdi Benchoufi, Philippe Ravaud

**Affiliations:** 1 INSERM U1153, Paris, France; 2 Assistance Publique-Hôpitaux de Paris, Centre d’Epidémiologie Clinique, Hôpital Hôtel-Dieu, Paris, France; 3 Université Paris Descartes-Sorbonne Paris Cité, Paris, France; 4 French Cochrane Centre, Paris, France; 5 Department of Epidemiology, Mailman School of Public Health, Columbia University, New York City, USA

**Keywords:** Blockchain, Transparency, Reproducibility, Data sharing, Privacy

## Abstract

Reproducibility, data sharing, personal data privacy concerns and patient enrolment in clinical trials are huge medical challenges for contemporary clinical research. A new technology, Blockchain, may be a key to addressing these challenges and should draw the attention of the whole clinical research community.

Blockchain brings the Internet to its definitive decentralisation goal. The core principle of Blockchain is that any service relying on trusted third parties can be built in a transparent, decentralised, secure “trustless” manner at the top of the Blockchain (in fact, there is trust, but it is hardcoded in the Blockchain protocol via a complex cryptographic algorithm). Therefore, users have a high degree of control over and autonomy and trust of the data and its integrity. Blockchain allows for reaching a substantial level of historicity and inviolability of data for the whole document flow in a clinical trial. Hence, it ensures traceability, prevents a posteriori reconstruction and allows for securely automating the clinical trial through what are called Smart Contracts. At the same time, the technology ensures fine-grained control of the data, its security and its shareable parameters, for a single patient or group of patients or clinical trial stakeholders.

In this commentary article, we explore the core functionalities of Blockchain applied to clinical trials and we illustrate concretely its general principle in the context of consent to a trial protocol. Trying to figure out the potential impact of Blockchain implementations in the setting of clinical trials will shed new light on how modern clinical trial methods could evolve and benefit from Blockchain technologies in order to tackle the aforementioned challenges.

## Background

Fixing methodology issues is one of the great challenges in contemporary biomedical research. Indeed, lack of reproducibility, related to a wide range of scientific misconduct aspects, from errors to frauds, compromises the outcomes of a clinical study and undermines research quality. Lack of reproducibility has been extensively studied, and medical scientific publications have been found on the whole to be not reproducible: they are full of “bugs”. Ioannidis et al. estimated a rate of about 80% non-reproducible studies [1–3]. This rate may be related to several types of errors, misconduct or fraud. Improving quality of research by better reproducibility and empowering both researcher communities with secure data sharing and patient communities with tools guaranteeing their privacy are desirable goals that can be achieved in part with Blockchain technology [4, 5].

Blockchain can have a global impact on clinical research because it allows for tracking, sharing and caring for data. Indeed, it involves a decentralised secure tracking system for any data interactions that could occur in the context of clinical trials, with a peer-to-peer inclusive network that enables data sharing on the research side and ensures all the needed transparency and care for privacy concerns on the patient community side.

In turn, this system can lead to more trust in clinical research, whose credibility has been considerably undermined with repeated scandals in recent years [6, 7]. Blockchain technology can be considered a basis for improved clinical research methodology and a step toward better transparency to improve trust within research communities and between research and patient communities.

## What is Blockchain?

Historically, Blockchain is known to be the technology powering Bitcoin, as an open, distributed public ledger recording all the Bitcoin transactions in a secure and verifiable way, without the need for a third party to process payments. In this context, Blockchain can be considered a full history of banking transactions.

More generically, Blockchain is a huge, public, secure and decentralised datastore [8, 9] of ordered records, or events, called blocks. Each block contains a timestamp and is linked to a previous block [10]. Events can be updated by only a majority of users. Information cannot be erased. The datastore is owned by no one, is controlled by users and is not ruled by any trusted third party or central regulatory instance. In fact, trust is encoded in the protocol and maintained by the community of users.

In practice, the Blockchain architecture allows for storing proofs of existence of data. As the only proof of data is the data of proof, we believe that this is a paradigm shift for medical research methodology.

## Building reliable clinical studies: at each step, keep track and timestamp

Inviolability and historicity of data are two major features of data at the functional level, “the data level”. Regarding inviolability and historicity of data, it follows that Blockchain ensures that events are tracked in their correct chronological order, which largely prevents a posteriori reconstruction analysis.

**Fig. 1:**
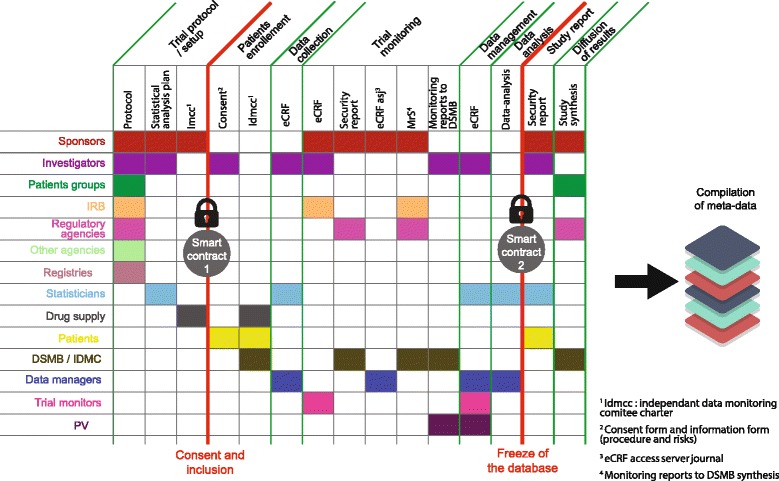
Clinical trial complex data workflow encoded in Blockchain

First, data integrity is ensured by the cryptographic validation of each transaction [11]. This is key to ensuring the sincerity of data — limiting data falsification, data “beautification” and in some sense data invention. Second, traceability and historicity of the data are among the core functionalities of the technology: each transaction with Blockchain is timestamped [12]. This information is publicly transparent; any user owns a copy of the proof of the time-stamped data. Figure 1 shows the complex flows of heterogeneous data and metadata that circulate in a clinical trial, implying numerous healthcare stakeholders, and all documents whose proof of existence can be stored in Blockchain. Thus, the existence of data becomes provable while the data remain confidential.

Below, we list non-exhaustive examples of key information that can advantageously “sit on the top” of the Blockchain:

- The data-sharing plan, including the schedule, dataset documentation and data-sharing agreement, if any, must be disclosed before the clinical trial begins, so this metadata can be timestamped in a chronological order in the unfalsifiable Blockchain.
- Before the clinical trial begins, consents and clinical trial protocol, including type of study, primary and secondary outcomes and inclusion and exclusion criteria, can be bundled into data structures stored on the Blockchain [13–15]. Data structures are then in one-to-one correspondence with consents and the protocol and its revisions, which accounts for robust proof of their existence. This feature can help prevent typical issues related to non-traceable clinical trial protocols, such as selective reporting outcomes related to selective reporting of harm, under-reporting of non-significant outcomes and mismatches between planned outcomes in the protocol and final publication. These issues are a well-documented source of bias [16–19]. In the Blockchain metadata set, we can also store information such as the mode of data collection, attribution method, dates of withdrawals to distinguish between early and late ones and dates of recurrent events.
- The statistical analysis plan is a critical need and is timestamped before the analysis is completed and, for a blinded study, before the data are unblinded. This plan includes the statistical methods, definition of harm events and multiple variable adjustments, if any. For example, sample size is a key item to compute to ensure that a study has enough power. Research teams often have no precise idea of the outcomes, so estimating the needed power in advance is difficult, which leads to an a posteriori calculus bias [20–22]. Here, we can imagine timestamping a set of metadata on the Blockchain: sample size, type I and type II errors, estimated event rate and treatment effect of interest. Timestamping will constitute a landmark in the Blockchain that will testify to the a priori-computed sample size.
- The analytical code [23] should be shared and made open to prevent analytical errors [24, 25]. Taking into account that scripts continue to evolve and that a fixed state of the code is used to process the data, this precise state of the code must be “frozen” and timestamped to ensure that conditions under which data were checked and analysed are reproducible. Numerous tools enable the collaborative sharing of different versions of the code: “git” is by far the most used. It provides for version control, but git (or any version control system such as mercurial or svn) cannot prevent a timestamp alteration [26]. The timestamp code on the Blockchain is, for todays’ state of technology, the only robust unalterable timestamping method.

## Privacy by design and data sharing in community-driven medicine

At the experience level, “the community level”, Blockchain is sometimes described as “trustless”, which can offer the right conditions for data sharing. In fact, trust is built inside the protocol. Blockchain can be considered as a “privacy-by-design” peer-to-peer infrastructure. With the level of trust it can enforce, it should be considered a path through the age of community-driven methodologies. Polls consistently show that about 80% of consumers are eager to share their medical information [27], provided privacy and security can be ensured. With the transparency of the Blockchain database — owned by no one, publicly writable by anyone and with strong crypto-oriented consistency of the database transaction — users do not need any third party to trust the system. Thus, the database opens a wide path to the data user’s control or differential privacy, data sharing and community-driven clinical study [23]: in a trusted environment, clinical research teams can “crowd-recruit” people to be enrolled in protocols with the help of community management techniques, and people can also volunteer to participate in such studies. Indeed, the Estonian e-Health authority has just implemented a Blockchain solution enabling storage of a million health records, letting patients control data access through a “Keyless Signature Infrastructure” [28, 29].

On the researcher side, data sharing is a subject of great interest and can provide many benefits. Indeed, sharing anonymised raw data, analysable datasets or a statistical analysis plan is a strengthening factor for reproducibility in science, opening clinical trials to secondary analysis or meta-analysis [24, 30–32]. Blockchain implementations can enable distributed, secure cloud data sharing. The advanced Massachusetts Institute of Technology (MIT) project Enigma, still under testing and not officially released, is most promising. Enigma’s Blockchain approach enables secure data sharing on a large scale and on a perimeter, finely controllable by the user who is sharing the data. With this kind of implementation, the data can be shared among any users or group of users, whether investigators, publishers or patients. The idea behind the technique is differential privacy: the user can fine-tune the equilibrium dose between publicly transparent data and control of the shared part between approved entities. Blockchain enables differential privacy in a secure way.

## Clinical trial phase control: Smart Contracts

Besides archiving clinical trial-phase-compilable metadata on the Blockchain, we can also chain together different clinical trial steps so that each step depends on its predecessor. Blockchain technologies bring tools to achieve these “slicing” and “chaining” processes, called Smart Contracts, and can enforce the level transparency, traceability and control over clinical trial sequences.

According to Wikipedia, “Smart Contracts are computer protocols that facilitate, verify, or enforce the negotiation of a contract” [33], and their execution can be implemented by using cryptographic hash chains. Practically, Smart Contracts enable the validation of a step with the only condition that every preceding step has been fully validated. For example, the chain of successive blocks could verify that the designed methodology has been followed, and the material presented to publishers would consist of the publication itself and the set of blocks that constitute the Smart Contract, whose correct execution indicates proof that the study was well conducted.

Figure 1 shows that the Smart Contract represents a piece of code that holds a programmatically written contract between as many parties as needed, without any trusted third party, and that executes algorithmically according to the terms provided by the contracting parties. Examples of Smart Contracts are allowing for patient inclusion with the only condition that they have consented or for enabling data analysis with the only condition that the database is frozen. Each of the clinical trial steps detailed in the figure can be chained together in a preceding order, consolidating a transparent trial and preventing a posteriori reconstruction or beautification of data.

## A proof of concept for collection of consent

In a proof-of-concept experimental study, we implemented a Blockchain system to collect participant consent for a clinical trial ([34] (under review), [35]). Indeed, the US Food and Drug Administration reports that almost 10% of the trials that they monitor feature issues related to consent collection: failure to obtain written informed consent, unapproved forms, invalid consent document, failure to re-consent to a revised protocol and missing institutional review board approval to protocol changes [36, 37]. Precisely, in a fake experimental study, we timestamped each patient consent on the Blockchain and asked again for consent renewal with each revision of the protocol. We obtained a unique master document that holds, in a single data structure or piece of code called Chainscript [38], all the consent collection data, each bound to a version of revised protocol versions. In fact, these data are “hashed”, that is, formatted into a sort of cryptographic form of the real consent and protocol document data. Of importance, this master document represents a secure, robust proof of existence of the whole consent-collection process because of a strict one-to-one correspondence between hashed data and effective consent data. Also, this proof of existence can be checked on any dedicated public website.

## Conclusions

Blockchain technology is a major opportunity for clinical research: it can help in structuring more transparent checkable methodology and, provided a set of core metadata is defined, can help check clinical trial integrity, transparently and partly algorithmically. Ultimately, Blockchain can lead to the structuration of some kind of community-driven Internet of health data, gathering researchers and patient communities, social networks and Internet of Things data flows, at a global dimension, with features of individual granularity, decentralisation and security and with transparent interactions to ensure easier and more transparent analysis.

## Glossary

Bitcoin: A cryptocurrency and a payment system running on a peer-to-peer decentralised network, called Blockchain, enabling transactions between users without any third-party approval. Any of these transactions are stored on a shared datastore called a Ledger and validated through a complex cryptographic system.
Blockchain: A peer-to-peer network of users sharing a datastore. This datastore is the assembly of blocks of data, linearly chained together using cryptographic signature. The chaining process is validated by each node of the network.
Hash: A mathematical process to generate a fixed-length string of data from variable-length data.
Ledger: The public datastore, shared by all the users. It is an “append-only” datastore in that its restricted usage allows data to be stored only in an immutable way.
Node: Designates any computer connected to the Blockchain network.
Smart Contract: A piece of code that allows automatic transactions with the Ledger. This program executes when any general-purpose conditions defined by the contracting parties are met.
Transaction: An asset transfer between contracting parties, whose trace is registered in the Ledger.

## References

[1] Ioannidis JP (2005). Why most published research findings are false. PLoS Med.

[2] Colhoun HM, McKeigue PM, Davey Smith G (2003). Problems of reporting genetic associations with complex outcomes. Lancet.

[3] Ioannidis JP (2003). Genetic associations: False or true?. Trends Mol Med.

[4] Blockchain for Open Science and Knowledge Creation, Bartling, Sönke, & et contributors to living document. 2017. https://docs.google.com/document/d/1Uhjb4K69l0bSx7UXYUStV_rjuPC7VGo0ERa-7xEsr58/edit.

[5] Irving G, Holden J (2016). How blockchain-timestamped protocols could improve the trustworthiness of medical science [version 1; referees: 2 approved]. F1000Research.

[6] http://retractionwatch.com/category/by-author/don-poldermans/.

[7] https://fr.wikipedia.org/wiki/BIA_10-2474.

[8] Blockchain definition. https://en.bitcoin.it/wiki/Block_chain. Accessed 20 Dec 2016

[9] Understanding Modern Banking Ledgers through Blockchain Technologies: Future of Transaction Processing and Smart Contracts on the Internet of Money Gareth W. Peters, Efstathios Panayi, CoRR abs/1511.05740. 2015. http://arxiv.org/pdf/1511.05740.pdf.

[10] https://en.wikipedia.org/wiki/Blockchain.

[11] Bitcoin and Decentralized Trust Protocols, Ricardo Pérez-Marco (CNRS, Univ. Paris 13, Paris, France). 2016. http://arxiv.org/pdf/1601.05254.pdf.

[12] Decentralized Trusted Timestamping using the Crypto Currency Bitcoin, Bela Gipp, Norman Meuschke, André Gernandt National Institute of Informatics Tokyo, Japan.

[13] CONsolidated Standards of Reporting Trials Guidelines, (Consort Guidelines). http://www.consort-statement.org/consort-2010.

[14] Chan AW, Tetzlaff JM, Altman DG, Dickersin K, Moher D (2013). Spirit 2013: New guidance for content of clinical trial protocols. Lancet.

[15] WHO Trial Registration Data Set (Version 1.2.1), International Clinical Trials Registry Platform (ICTRP). http://www.who.int/ictrp/network/trds/en/. Accessed 20 Dec 2016.

[16] Ioannidis JP, Evans SJ, Gotzsche PC, O’Neill RT, Altman DG (2004). Better reporting of harms in randomized trials: An extension of the CONSORT statement. Ann Intern Med.

[17] International Conference on Harmonisation E9 Expert Working Group (1999). ICH Harmonised Tripartite Guideline. Statistical principles for clinical trials. Stat Med.

[18] Moher D, Cook DJ, Eastwood S, Olkin I, Rennie D (1999). Improving the quality of reports of meta-analyses of randomised controlled trials: The QUOROM statement. Quality of Reporting of Metaanalyses. Lancet.

[19] Stroup DF, Berlin JA, Morton SC, Olkin I, Williamson GD, et al. A proposal for reporting. Meta-analysis of Observational Studies in Epidemiology (MOOSE) group. JAMA. 2000;283:2008–12. 10.1001/jama.283.15.2008 10789670

[20] Freiman JA, Chalmers TC, Smith H, Kuebler RR, Bailar JC, Mosteller F (1992). The importance of beta, the type II error, and sample size in the design and interpretation of the randomized controlled trial. Medical uses of statistics.

[21] Moher D, Dulberg CS, Wells GA (1994). Statistical power, sample size, and their reporting in randomized controlled trials. JAMA.

[22] Mulward S, Gøtzsche PC (1996). Sample size of randomized double-blind trials 1976-1991. Dan Med Bull.

[23] Sandve GK, Nekrutenko A, Taylor J, Hovig E (2013). Ten simple rules for reproducible computational research. PLoS Comput Biol.

[24] Giles C. Financial Times. 2014. [October 17, 2014]. (Thomas Piketty's exhaustive inequality data turn out to be flawed). http://www.ft.com/cms/s/0/c9ce1a54-e281-11e3-89fd-00144feabdc0.

[25] IOM (Institute of Medicine) (2012). Evolution of translational omics: Lessons learned and the path forward.

[26] Git documentation. https://git-scm.com/. Accessed 25 Jan 2017.

[27] Chu, S. Apple watch release news: survey finds 80 percent of US employees would give health data from wearables to employers. iDigitalTimes (2 February; Accessed 7 July 2015). http://www.idigitaltimes.com/apple-watch-release-news-survey-finds-80-percent-us- employees-wouldgive-health-data-411578.

[28] Buldas A, Kroonmaa A, Laanoja R. Keyless Signatures’ Infrastructure: how to build global distributed hash-trees. https://eprint.iacr.org/2013/834.pdf.

[29] Guardtime secures over a million Estonian healthcare records on the blockchain. http://www.ibtimes.co.uk/guardtime-secures-over-million-estonian-healthcare-records-blockchain-1547367. Accessed 25 Jan 2017.

[30] Sharing Clinical Trial Data: Maximizing Benefits, Minimizing Risk. Committee on Strategies for Responsible Sharing of Clinical Trial Data; Board on Health Sciences Policy; Institute of Medicine. Washington, DC: National Academies Press; 2015. 25590113

[31] IOM. Workshop on principles and best practices for sharing data from environmental health research. Washington, DC; 2014.

[32] Jasny BR (2011). Again, and again, and again…. Science.

[33] https://en.wikipedia.org/wiki/Smart_contract.

[34] Benchoufi M, Porcher R, Ravaud P (2017). Blockchain protocols in clinical trials: Transparency and traceability of consent. F1000Research.

[35] Nugent T, Upton D, Cimpoesu M (2016). Improving data transparency in clinical trials using blockchain smart contracts [version 1; referees: 3 approved]. F1000Research.

[36] Office of Scientific Investigations, Metrics (2014). US Food and Drug Administration.

[37] Barney JR, Antisdel M (2013). Common problems in informed consent. Human Research Protection Program (HRPP).

[38] Chainscript documentation. Chainscript is developed by a Blockchain solutions provider, Stratumn SAS. http://chainscript.io. Accessed 25 Jan 2017.

